# Fish Oil Supplement Mitigates Muscle Injury In Vivo and In Vitro: A Preliminary Report †

**DOI:** 10.3390/nu16203511

**Published:** 2024-10-16

**Authors:** David W. Russ, Courtney Sehested, Kassidy Banford, Noah L. Weisleder

**Affiliations:** 1 School of Physical Therapy & Rehabilitation Sciences, Morsani College of Medicine, University of South Florida, Tampa, FL 33612, USA; courtney.sehested@gmail.com; 2 Department of Physiology, Ohio State University College of Medicine, Columbus, OH 43210, USA; kassidybanford@gmail.com; 3 Department of Molecular & Cellular Biochemistry, University of Kentucky, 741 South Limestone Street, BBSRB 143, Lexington, KY 40536, USA; nlwe230@uky.edu

**Keywords:** aging, contractility, membrane injury, eicosapentaenoic acid, docosahexaenoic acid, sarcopenia

## Abstract

**Background:** Following injury, older adults exhibit slow recovery of muscle function. Age-related impairment of sarcolemmal membrane repair may contribute to myocyte death, increasing the need for myogenesis and prolonging recovery. Dietary fish oil (FO) is a common nutritional supplement that may alter plasma membrane composition to enhance the response to membrane injury. **Methods:** We assessed effects of an 8-week dietary intervention on muscle contractile recovery in aged (22 mo.) rats on control (n = 5) or FO (control + 33 g/kg FO (45% eicosapentaenoic acid; 10% docosahexaenoic acid); n = 5) diets 1-week after contusion injury, as well as adult (8 mo., n = 8) rats on the control diet. **Results:** Recovery was reduced in aged rats on the control diet vs. adults (63 vs. 80%; *p* = 0.042), while those on the FO diet recovered similarly to (78%) adults. To directly assess sarcolemma injury, C2C12 cells were cultured in media with and without FO (1, 10, and 100 μg/mL; 24 or 48 h) and injured with an infrared laser in medium containing FM4-64 dye as a marker of sarcolemmal injury. FO reduced the area under the FM4-64 fluorescence-time curve at all concentrations after both 24 and 48 h supplementation. **Conclusions:** These preliminary data suggest FO might aid recovery of muscle function following injury in older adults by enhancing membrane resealing and repair.

## 1. Introduction

Although older adults (OAs) tend to participate less often in occupational and athletic activities typically associated with mechanical muscle injury, they are still at risk. Many OAs initiate exercise and mobility programs [1] that carry a risk of injury and falling; the most common mechanism of injury in OAs [2,3] carries a risk of muscle contusion [3,4,5,6]. Geriatric clinical care following falls understandably focuses on fractures [4], but muscle contusion can reduce function and mobility, exacerbating physical inactivity in the absence of fracture [5]. Even young adults can take weeks to fully recover from a muscle contusion [7,8]. However, contusion injury has not received much study in aging models. We have previously reported that the magnitude of acute functional muscle impairment following contusion was similar in adult and aged rats [9] but have not examined short- or long-term recovery from contusion.

Standard treatment of contusion involves non-steroidal anti-inflammatory drugs (NSAIDS), mostly for pain control. However, the direct benefits of NSAIDs to injured muscles remain unclear, especially after the acute phase [10]. Some data suggest they might even impair recovery [11], though the extent of this negative impact is equivocal [12,13]. Dietary supplements, such as fish oil (FO), might offer effective alternatives to, or additional benefits in combination with, standard drug treatments. Multiple beneficial effects on OAs have been ascribed to dietary FO, many linked to anti-oxidant and anti-inflammatory actions [14,15,16]. Indeed, a scoping review identified FO as one of only 16 micronutrient supplements with potential to improve musculoskeletal health in aging [17]. Moreover, unlike several other dietary supplements, FO does not appear to blunt muscle responses to exercise [18] and would thus be potentially equally useful in active and sedentary older adults. Although the anti-coagulant effects attributed to FO could conceivably worsen contusion injury, we have found that FO neither reduced nor increased the acute functional impairment following muscle contusion in adult and aged rodents [9].

Incomplete recovery from muscle injury is a frequently-reported consequence of old age [19,20,21,22,23] and has been observed even if the degree of acute injury is equal [23]. Muscle cells are exposed to regular mechanical injury during contraction and thus need a robust, rapid process to maintain sarcolemmal membrane integrity and maintain cell homeostasis. Inhibition of sarcolemmal repair processes can increase the muscle degeneration and resulting fibrosis [24,25,26], thus impairing and/or slowing recovery. We have previously reported that aged muscle exhibits increased content of dysferlin [27], a protein responsive to membrane injury [28,29], suggesting that this increased expression in aged muscle could be compensation for more frequent or long-lasting membrane stress in old age. Dysferlin is known to act in concert with Tripartite motif-containing protein 72 (TRIM72/MG53) to repair membrane disruption in response to injury [30], but we are unaware of any reported age-related differences in TRIM72/MG53. It has been shown that aging affects the lipid composition of cell membranes, which could impair the muscle membrane resealing process [25]. Dietary FO can also affect membrane lipid metabolism and composition [31,32] and thus might counteract age-related changes and, at least partially, restore recovery following injury. Thus, while FO did not provide prophylactic protection against injury in our earlier study [33], it might still enhance or accelerate recovery from injury, possibly by enhancing membrane repair/resealing.

In this preliminary report, we evaluated the effects of FO subacute (7-day) functional (i.e., contractile) recovery of in situ muscle contractility in aged rats. Because impaired recovery from injury has been reported with increased age, we also tested a group of adult rats on a standard diet compared to aged rats on the standard diet or one supplemented with FO. In addition, we examined the effects of age, injury, and FO on the abundance of the membrane repair proteins dysferlin and TRIM72/MG53. We also conducted an in vitro assay of direct membrane injury and resealing of myoblasts incubated with the same FO used in the in situ studies. Because poor membrane repair would be expected to result in greater injury, we also evaluated a marker of the endoplasmic/sarcoplasmic reticulum stress response. ER/SR stress is associated with atrophy and weakness during muscle disuse (i.e., hindlimb suspension) [34]. Among the factors in this stress response is the unfolded protein response, which involves molecular chaperones working to appropriately refold proteins damaged by injury [35]. Accordingly, we also probed the muscles tested in situ for 78 kD glucose-related protein (Grp78, A.K.A. BiP), a common marker of ER stress [35,36]. We hypothesized that FO supplementation would improve contractile recovery in aged rats to a level similar to that seen in adult animals and that FO would enhance the membrane resealing process.

## 2. Materials and Methods

### 2.1. In Vivo Testing

Experimental animals: Adult (6–8 months; n = 8) and aged (20 months; n = 10), male and female Sprague Dawley rats were purchased from Envigo (Indianapolis, IN, USA). All aged rats were acclimated for 1 week prior to assignment to the experimental diet group, with ad libitum access to standard natural chow (Envigo #T8640 Teklab 22/5, Madison, WI, USA) and water, while adult rats were maintained on the control diet throughout the study. All animal use and procedures were approved by the University of South Florida IACUC (approval code: 8039; approval date: 30 November 2020), and “Principles of laboratory animal care” (NIH publication No. 86-23, revised 1985) were followed throughout the study.

Dietary intervention in aged animals: Following acclimation, aged rats were balanced by body mass and food intake, and equal numbers in each age group were assigned to control (Ctl) (AIN-93M purified diet, Research Diets, New Brunswick, NJ, USA) or experimental (FO) diet (AIN-93M formulated with 33 g/kg commercial fish oil product (EPAX 4020TGN, EPAX, Ålesund, Norway AS; 10% docosahexaenoic acid (DHA) and 45% eicosapentaenoic acid (EPA)). Diets were maintained for 8 wks.

Injury protocol: After 8 wks, all animals were anesthetized with isoflurane, and the left hindlimb was placed in a custom injury apparatus we have described and utilized previously [9]. Muscle injury was produced by dropping a known weight and a known height onto a custom impactor placed on the medial gastrocnemius muscle (MG). Following injury, 1.0 mg/kg sustained-release buprenorphine was administered via subcutaneous injection. A second injection was administered 24 h later.

Grip strength: Bilateral forelimb grip strength was determined as previously described [37], using a Columbus Instruments 1027DR grip strength meter (Columbus, OH, USA) on Days 1 and 32 of the dietary intervention in aged rats only, as our previous study suggested that FO might slow the decline of grip strength in aging [33]. Peak forces of 5 trials were averaged for our measure of grip strength and were expressed in absolute terms and relative to body mass.

### 2.2. In Situ Testing

Muscle contractility: Prior to contractile testing, animals were anesthetized by injection of ketamine:xylazine (40:10 mg kg^−1^ body mass; IP). In situ contractility of the MG was assessed 7 d post-injury in the injured and uninjured hindlimbs of all rats using our published experimental setup [9] and the Aurora Muscle Testing System (Ontario, Canada). Peak force responses to single-pulse and 6-pulse (100 Hz), 200 µs pulse duration, and stimulation trains delivered to the sciatic nerve were evaluated because studies of awake moving rats show that most motor units fire in short (≤6 spikes) bursts [38]. Following testing, animals were euthanized by intracardiac injection (Euthasol, 100 mg kg^−1^). The ratio of force production by the injured MG to that of the uninjured MG was used as the index of recovery of muscle function.

Immunoblotting: The abundance of proteins associated with membrane injury was determined via Western blotting of injured and uninjured muscle tissue. Portions of the MG muscle were homogenized as described previously [27]. Proteins were separated by SDS-PAGE using 8–15% gels, depending on the weight of the protein of interest, then transferred overnight to PVDF membranes. After drying for 24 h, membranes were blocked and incubated overnight with at 4 °C primary antibodies to Dysferlin (Sigma-Aldrich, St. Louis, MO, USA, SAB4200453) and Trim72/MG53 (custom antibody [39]). The next day, blots were rinsed five times in TBS-T, incubated with secondary antibodies (LiCor) at room temperature for 2 h, rinsed 5 times with TBS-T, and scanned on an Odyssey fluorescence scanning system (LiCor, Lincoln, NE, USA). An additional protein, Grp78, which is associated with ER stress, and the unfolded protein response were also assessed using the same procedures with antibodies (Sigma-Aldrich SAB4501452). We have previously found that this protein is elevated in acutely injured muscle regardless of age [9]. Proteins were visualized and analyzed by densitometry using the scanner manufacturer’s software (Image Studio 5.2, LiCor, Lincoln, NE, USA). Membranes were later stripped and stained for total with Coomassie Brilliant Blue R25. Band intensities were normalized to total protein per lane.

### 2.3. In Vitro Testing

Cell culture conditions and fish oil treatment: C2C12 mouse myoblast cells (American Type Culture Collection, Manassas, VA, USA) were maintained in a humidified environment at 37 °C and 5% CO_2_ in Dulbecco’s modified Eagle’s medium (ThermoFisher, Waltham, MA, USA), supplemented with 10% fetal bovine serum, 100 units/mL penicillin, and 100 μg/mL streptomycin. C2C12 myoblasts were grown to 70% confluence on 35 mm glass-bottomed petri dishes (MatTek, Ashland, MA, USA) before the culture media was supplemented with various doses of fish oil (1 μg/mL, 10 μg/mL, and 100 μg/mL) for either 24 or 48 h.

Plasma membrane repair assay: The plasma membrane repair capacity of C2C12 cells was measured using laser scanning confocal microscopy, essentially as previously described [40]. In brief, C2C12 cells were cultured on 35 mm glass-bottomed petri dishes (MatTek, Ashland, MA, USA) and then washed and transitioned into modified Tyrode’s solution (140 mM NaCl, 5 mM KCl, 2 mM MgCl2, and 10 mM HEPES (pH 7.2). Membrane injury was induced using a FluoView FV1000 multi-photon confocal laser-scanning microscope (Olympus, Center Valley, PA, USA) on cells in Tyrode’s solution supplemented with 2.5 µM FM4-64 dye (Invitrogen, Eugene, OR, USA). A circular region of interest was selected along the edge of the plasma membrane and irradiated at 50% of maximum infrared laser power for 5 s. Pre- and post-damage images were captured every 3 s for a total of 60 s. The extent of membrane damage was analyzed using Fiji ImageJ software, Version 20170530 (National Institute of Health, Bethesda, MD, USA), measuring the fluorescence intensity encompassing the site of damage with results represented as ∆F/F0, as previously described [41]. Briefly, the fluorescent intensity at the injury site is measured, and then the fluorescence intensity level in the injury area before the injury is subtracted from that value. The remaining signal is divided by the fluorescence intensity level in the injury area before the injury. This calculation is repeated in each image captured over the 60 s time course. These ∆F/F0 results are plotted over time, and the area under the curve (AUC) was calculated using GraphPad Prism Version 10 (GraphPad Software, La Jolla, CA, USA) to provide a measurement of the total dye entry when the cell is injured.

### 2.4. Statistical Analysis

Group differences in initial body mass, food consumption, and food consumption normalized to body mass were initially analyzed by ANOVA, followed by ANCOVA with sex as a covariate. The percent changes over 8 weeks were assessed similarly. Group differences in the ratio of injured/uninjured forces were evaluated using the Kruskall–Wallis test. In the event of a significant omnibus test, post hoc testing was conducted via the Dunn–Bonferroni test. Grip strength was assessed using Wilcoxon signed-rank tests to compare differences within dietary conditions, and Mann–Whitney U-tests were used to compare differences across groups. Immunoblot data were analyzed by 3 × 2 (Group X Injury), with injury as a repeated factor. Post hoc testing in the case of significant main effects or interactions was conducted with Fisher’s LSD test. For the in vitro experiments, one-way ANOVAs were used to assess peak FM4-64 intensity and AUC for the 24 and 48 h incubation conditions, separately. Analyses were performed in SPSS (version 29, IBM Corp., Armonk, NY, USA)

## 3. Results

### 3.1. In Vivo Testing

Animal characteristics: Animal weights and food consumption (disappearance) are presented in Table 1. Based on these data, the average EPA dosage was 0.540 g/kg bw/day, while the DHA dosage in the aged FO group was 0.112 g/kg bw/day, calculated as we have previously performed [9]. Although there was no significant effect of group for initial body masses and rates of food disappearance (*p* = 0.547 and 0.219, respectively), differences emerged upon inclusion of sex as a covariate in the analysis due to the unbalanced distribution of males and females across groups (*p* = 0.001 and 0.002, respectively). The initial food consumption normalized to body mass exhibited no significant effects for either analysis. Similarly, none of the percent changes in these variables showed a significant effect of group.

**Figure 1:**
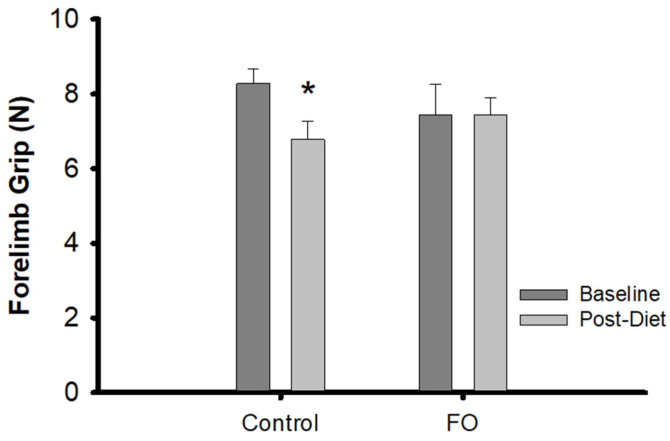
Changes in forelimb grip strength: Baseline and post-dietary intervention bilateral forelimb grip breaking force in aged rats on the control and FO diets. * = significant within group difference by Wilcoxon signed-rank tests (*p* = 0.028).

Grip strength: There were no differences in grip strength between the aged animals on the control and on the experimental diets. However, within-groups testing showed a significant (*p* = 0.028) decline over the 8-week dietary intervention in the aged rats on the control diet, but not the experimental diet (Figure 1).

### 3.2. In Situ Testing

**Figure 2:**
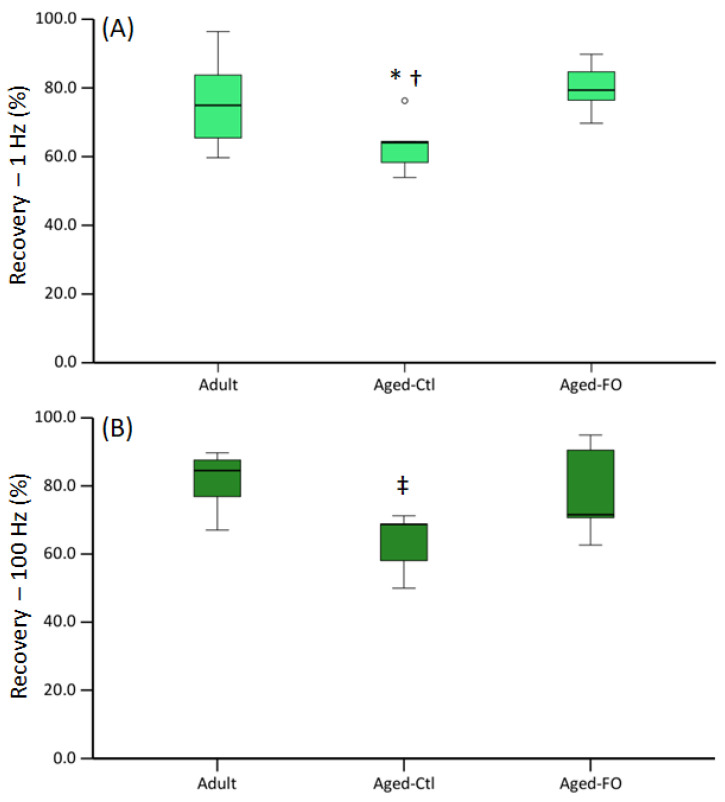
Muscle force at 7 d post-injury: Injured/uninjured peak force in response to (**A**) 1 Hz and (**B**) 100 Hz stimulation. Data are presented as boxplots showing median, IQR and CI. Kruskall–Wallis test showed significant group effect for 1 and 100 Hz (*p* = 0.050 and 0.040, respectively). * = significantly different from aged-FO; ^†^ = trend for difference from adult (*p* = 0.080), ^‡^ = significantly different from adult (*p* = 0.042).

Muscle contractility: At 7 d post-injury, there was a significant effect of group on the ratio of injured/uninjured force in response to 1 and 100 Hz stimulation, with the aged/control diet group showing the least recovery of force (Figure 2).

**Figure 3:**
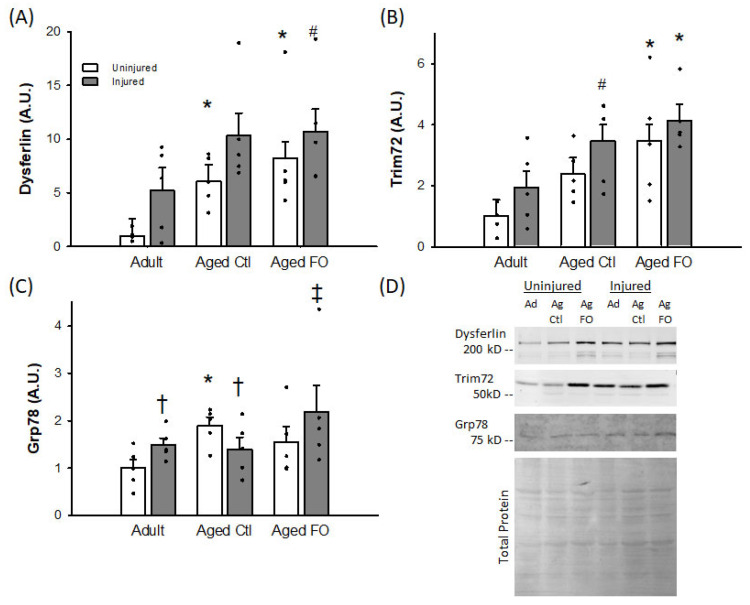
Mean (+SE) protein abundance in injured and uninjured muscles. Values are normalized to uninjured Ad means (arbitrary units, A.U.). (**A**) Dysferlin, (**B**) Trim72/MG53, (**C**) Grp78 and (**D**) representative blots for Dysferlin, Trim 72/MG53, and Grp78, along with total protein staining. Ad = adult; Ag Ctl = aged control diet; Ag FO = aged FO supplemented diet; n = 5/group. *  =  significantly different from corresponding Ad group (*p* < 0.050); # = trend for difference from corresponding Ad group (0.050 < *p* < 0.100); ^‡^ = significantly different from corresponding uninjured group (*p* < 0.050); ^†^ = trend for difference from corresponding uninjured group (0.050 < *p* < 0.100).

*Immunoblotting*: Dysferlin and Trim72/MG53 exhibited similar patterns of expression with a significant main effect of group (*p* = 0.006 and 0.010, respectively), due to increased levels in the aged groups relative to the young control animals (Figure 3A,B). Both proteins also exhibited an increase in response to injury, though this effect was significant for Dysferlin (*p* = 0.040) but not Trim72/MG53 (*p* = 0.053). No group X injury interaction was observed for either protein. In contrast to these membrane repair proteins, Grp78 did exhibit a significant group X injury interaction (*p* = 0.021), with no individual main effect for either factor. The interaction was largely driven by the aged-control diet group, which was increased in uninjured muscles but reduced in the injured muscles relative to the other two groups (Figure 3C).

### 3.3. In Vitro Testing

**Figure 4:**
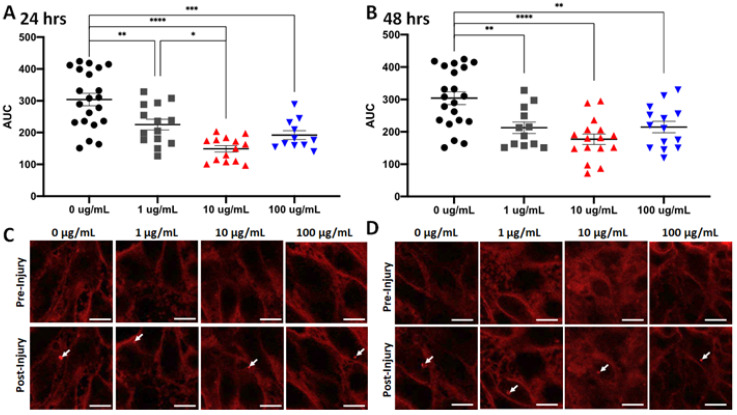
Fish oil exposure increases membrane repair in cultured skeletal myoblasts. (**A**) Fish oil was supplemented into the culture media for C2C12 cells at various concentrations (1 μg/mL, 10 μg/mL, and 100 μg/mL) for 24 h before the cells were subjected to laser injury in the presence of FM6-64 dye. FM-464 fluorescence signal at the laser injury were recorded by confocal microscopy for 60 s and then the area under curve (AUC) was determined for curves of the changes in fluorescent signal over time. Means of each treatment group were compared by one-way ANOVA with significance presented by * = *p* < 0.05, ** = *p* < 0.005, *** = *p* < 0.001, and **** = *p* < 0.0001. Data are represented as means ± SEM. (**B**) Similar results were seen in identical assays with cells exposed to fish oil for 48 h. (**C**) Representative images of C2C12 cells exposed to fish oil for 24 h before injury (top) or 60 s post-injury (bottom). Arrows indicate sites of laser injury. Scale bar represents 10 μm. (**D**) Representative images of C2C12 cells exposed to fish oil for 48 h before injury (top) or 60 s post-injury (bottom). Arrows indicate sites of laser injury. Scale bar represents 10 μm.

Plasma membrane repair assay: Generally, C2C12 cells incubated in the FO exhibited reduced AUC and peak FM4-64 fluorescence intensity at areas of membrane disruption following laser injury compared to those incubated in the control medium. There were some effects from dosage, as incubation in 10 µg/mL showed a greater effect than 1 µg/mL, but no additional effect was seen for 20 µg/mL. Of note, increasing the incubation time from 24 to 48 h did not appreciably change the results (Figure 4). These results suggest that FO enhances membrane resealing in the muscle-derived C2C12 cells.

## 4. Discussion

The principal findings of this preliminary study were that an 8-week FO-supplemented diet improved muscle contractile function 7 days post-contusion injury and that the same FO product added to the incubation medium of C2C12 cells enhanced membrane resealing following laser injury of the plasmalemma.

Our previous study found that both adult and aged MG muscle exhibited similar (~45%) loss of contractile function acutely following contusion injury, with or without a FO-supplemented diet [9]. In the present study, adult MG muscles at 7 days post-injury exhibited 20–25% reduction of force vs. uninjured muscles, similar reports of contractility 7 days following a single, mechanical injury [7,42] that induced a comparable level of acute contractile impairment to that seen with our contusion protocol [9]. In contrast, the aged MG muscles from rats on the control diet exhibited a 30–40% reduction of force, while the aged MG muscles from rats on the experimental diet exhibited a reduction of force similar to that of the adult MG. These data suggest that increased age slows recovery of contractile function following contusion injury and that an 8-week FO-supplemented diet largely offsets this impairment.

The age-associated increase in dysferlin in uninjured muscles is similar to that which we have reported previously [27], and the observation of increased dysferlin abundance 1 week post-contusion is consistent with data from gastrocnemius muscles of young mice following electroporation injury [43]. It has been suggested that this sustained increase following acute injury may potentiate the membrane-sealing response to future injuries, which one might speculate are more likely in a previously-injured muscle. However, the similar pattern of expression seen in aged rats on the control and FO diets suggests that an effect of FO on dysferlin does not account for the enhanced recovery following injury. While we are unaware of studies reporting TRIM72/MG53 expression in the subacute and chronic injury phases, it seems reasonable that this protein would increase similarly to dysferlin, given the coordinated function of the two proteins in response to injury. It has also been reported that activation of AMPK is important for the membrane repair function of dysferlin, as rescued muscle phenotype in models of dysferlinopathy and enhanced myotube membrane recovery from injury, even when dysferlin levels were unchanged [44]. It could be that changes in p-AMPK/AMPK with FO could account for the improved contractile recovery despite the lack of effect on dysferlin. However, it is worth noting that aging is not a dysferlinopathy per se. Moreover, AMPK hyperphosphorylation has been reported in aging rat muscles [45], so a role for AMPK in aging membrane repair is not clear. Nevertheless, some [46,47,48], though not all [49,50,51], studies suggest that DHA and EPA can increase AMPK phosphorylation and activity, making this an appropriate target for future research.

The Grp78 response deviated somewhat from those of dysferlin and TRIM72/MG53. While aged muscle showed increased Grp78, consistent with previous observations [36,52], the injured muscles showed a different pattern than the membrane resealing proteins. We have previously shown that Grp78 is increased following acute contusion injury in adult and aged muscles [9], and elevated Grp78 has been reported 1 week following cardiotoxin-induced injury in young mice [53]. However, elevated Grp78 was seen in injured muscles of the adult and aged FO-supplemented rats, but not those in the aged control-diet group (Figure 3). Though associated with the ER stress and unfolded protein response resulting from proteins damaged by injury, Grp78 is also related to the chaperoning of newly synthesized proteins during tissue growth and regeneration [54,55]. It may be that the reduced Grp78 observed in the aged/control diet-fed rats compared to the adult and aged/FO diet-fed rats indicates an attenuated muscle regeneration with reduced new protein synthesis, contributing to the impaired functional recovery. It has also been reported that aging muscles contain tubular aggregates, abnormal cytoplasmic accumulations of membranes, typically enriched in SR markers [56]. Other investigators have reported that tubular aggregates also contain dysferlin and Grp78 [57]. A similar intramuscular aggregation pattern of TRIM72/MG53 has been reported in a mouse model of ALS with fragile membranes, though it was not definitively tied to tubular aggregates [58]. It is thus possible that the age-related increases seen in all three proteins evaluated in this study reflect increased tubular aggregates in addition to accumulated and/or ongoing focal membrane injuries. It has been suggested that tubular aggregates may be broken by degeneration/regeneration following significant muscle injury [59]. The differential changes in Grp78 and dysferlin (Figure 3) observed here suggest that a blanket change in tubular aggregates with injury is unlikely and an interaction between changes in tubular aggregates and other sites of protein localization is probably occurring.

Cultured C2C12 cells that received the same FO in their bathing medium exhibited a dose-dependent increase in membrane resealing following laser-induced membrane injury compared to those in medium without FO supplementation. These findings suggest a possible mechanism of action for the in vivo responses described above, since it has been observed that reduced sarcolemmal membrane repair increases susceptibility to mechanical muscle injury [29,39,40]. Though speculative, if membrane resealing is impaired in aged muscles and if dietary FO can correct that impairment, it could explain our in vivo findings. It is known that membrane composition and environment can affect its function and that age-related changes in membrane lipid makeup are associated with increased membrane fragility [60]. Moreover, both injury and dietary interventions, including FO, can alter membrane composition [31,61]. Thus, we speculate that changes in muscle membrane composition could explain the observed effects on membrane resealing observed in the current study. Further work is needed to explore these potential mechanisms in in vivo models of aging muscle injury.

The known antioxidant and anti-inflammatory effects attributed to FO might also speed recovery by, alone or in combination with effects on sarcolemmal repair, limiting the accumulation of chemical byproducts that could impair recovery. For example, it has been reported that reactive oxygen species induce greater inhibition of acetylcholine release at the neuromuscular junction of aged vs. adult rodents (NMJ) [62]. Thus, the anti-oxidant action of FO could limit this NMJ impairment post-injury and enhance recovery of contractile force. Interestingly, the decline in grip strength seen over 8 wks in the aged, control-fed rats was not observed in the aged rats on the experimental diet, similar to what we have observed previously, though in that study, the effect of FO showed only a trend for an effect on maintaining grip strength [9]. Thus, FO might enhance other aspects of muscle health in aging beyond recovery from mechanical injury.

These preliminary data suggest a potential role for dietary FO in promoting recovery of aging skeletal muscle function following mechanical injury. In addition, they provide proof-of-concept for at least one mechanism (improved sarcolemmal membrane resealing) for post-injury benefits of FO. However, as a preliminary study, there are limitations that must be acknowledged. First, we evaluated contractility and direct membrane resealing in different preparations: whole muscle and C2C12 myoblasts, respectively. In addition, we evaluated muscle function at a single time point—1 week post-injury. While our earlier study using the same injury model showed no acute benefit of FO, it would still have strengthened the study had we been able to test muscle function acutely and sub-acutely as well as during more prolonged recovery. We also tested only a few candidate mechanisms related to sarcolemmal injury (i.e., dysferlin, TRIM72/MG53), as these are known to be directly related to the membrane injury response. However, having identified a beneficial effect on subacute muscle function (contractility), future studies can examine other potential mechanisms, including, but not limited to, AMPK, membrane lipid composition, sarcoplasmic reticulum function, and myogenesis. Finally, because our focus was on improving aged muscle function, we did not evaluate the effects of FO in young animals.

Muscle function declines even in healthy, uncomplicated aging, and below a certain threshold, the risk of disability increases [63], whereby impaired muscle recovery is thought to contribute to the “negative staircase” effect in aging physical function [64]. It has been frequently observed that OAs exhibit reduced resiliency to muscle injury and disuse compared to younger adults [19,21,65,66,67,68], even when the magnitude of injury is no greater [69,70,71,72,73]. Thus, it is likely that impaired recuperative capacity plays a substantial role in the development of age-related weakness and physical disability [64]. Treatments that could enhance recovery might thus have great potential to improve health and wellness in old age. Further work is needed to evaluate this potential in dietary FO, in particular the extent to which FO supplementation could be used as a therapeutic intervention acutely following injury or if it needs to be consumed regularly as part of the normal diet.

## 5. Conclusions

The addition of an FO supplement to a standard diet appears to benefit aging muscle in rats by increasing recovery of muscle contractility in the sub-acute phase following a contusion injury. The same FO supplement, when included in a cell culture model, enhances sarcolemmal membrane resealing following acute laser injury. These data suggest that regular inclusion of FO in the diet may promote recovery from mechanical injury by enhancing membrane repair processes, though other effects of FO may be at work. Further study of these phenomena is needed to better understand the potential benefits of FO for aging muscle.

## Figures and Tables

**Table 1:** Animal weights and food consumption (disappearance).

	Adult (4M/4F)	Aged (2M/3F)	Aged (2M/3F)
Body Mass and Food Consumption			
Diet:	Ctl	Ctl	FO
Week 1 body mass (g)	286.9 ± 95.2	325.1 ± 62.3	332.2 ± 70.3
Body mass change at week 8 (%)	3.3 ± 3.5	1.2 ± 5.0	2.5 ± 4.5
Week 1 food disappearance (g/week)	65.7 ± 20.3	79.3 ± 15.4	83.5 ± 17.0
Week 8 food disappearance (g/week)	−6.7 ± 3.8	−3.9 ± 13.6	−1.6 ± 8.7
Week 1 food disappearance/body mass	0.241 ± 0.009	0.246 ± 0.014	0.254 ± 0.017
Week 8 food disappearance/body mass	−9.6 ± 5.2	−4.7 ± 15.2	−3.6 ± 11.7

## Data Availability

The data that support the findings of this study are available from the corresponding author upon reasonable request.
